# Case Study: The U.S. National Alzheimer's Plan

**DOI:** 10.1002/alz70860_110734

**Published:** 2025-12-23

**Authors:** Jennifer Pollack

**Affiliations:** ^1^ Alzheimer's Association, Chicago, IL, USA

## Abstract

The United States released its National Alzheimer's Plan in 2012, and updates are issued annually. Originally set to expire in 2025, the U.S. Congress last year extended the Plan through 2035. This presentation will focus on the significance of the Plan, progress made to date, and potential areas of focus over the next ten years.

